# Padrão Eletrocardiográfico de Brugada – Dificuldades no Reconhecimento de uma Condição Potencialmente Letal

**DOI:** 10.36660/abc.20210596

**Published:** 2021-08-09

**Affiliations:** 1 Universidade de São Paulo Faculdade de Medicina Hospital das Clínicas HCFMUSP São PauloSP Brasil Instituto do Coração (InCor) - Hospital das Clínicas HCFMUSP - Faculdade de Medicina da Universidade de São Paulo, São Paulo, SP – Brasil.

**Keywords:** Síndrome de Brugada, Padrão Eletrocardiográfico de Brugada, Prevalência de ECG de Brugada, ECG Padrão de 12 Derivações, Brasil

O achado de uma peculiar elevação do segmento ST nas derivações precordiais direitas foi inicialmente considerado uma variante do normal.^1^ A primeira pista de que esse sinal eletrocardiográfico (ECG) e a morte cardíaca súbita (MSC) poderiam estar associados surgiu a partir de uma série de casos veiculada em 1989.^2^ Três anos depois, Brugada *et al*. publicaram um relato de oito pacientes que apresentavam bloqueio de ramo direito, elevação persistente do segmento ST em V1-3 e episódios múltiplos de fibrilação ventricular, uma nova síndrome clínica e eletrocardiográfica mais tarde batizada pelo sobrenome desses autores.^3^ Hoje, a síndrome de Brugada (SB) ainda atrai muito interesse devido à sua alta prevalência em regiões específicas do mundo e letalidade potencial em adultos jovens que, sob outros aspectos, seriam saudáveis.^4^^,^^5^

Responsável por 4% a 12% de todos os casos de MSC no mundo e por um quinto dos que acontecem em corações estruturalmente normais,^6^ a SBr é uma cardiopatia autossômica dominante, com penetrância incompleta quanto a sexo e idade, causada por canais de sódio disfuncionais.^7^^,^^8^ Embora geralmente silenciosa, a SB se destaca clinicamente pela predominância do sexo masculino, e manifestação entre a terceira e a quinta décadas de vida.^4^ Os pacientes acometidos exibem alterações eletrocardiográficas e maior suscetibilidade a arritmias cardíacas.^8^ Os sintomas, quando presentes, podem variar desde síncope até MSC, dependendo do tipo e da duração dos eventos arrítmicos, que quase sempre ocorrem em repouso ou condições vagotônicas.^8^

Os achados eletrocardiográficos incluem anormalidades de despolarização e repolarização ventricular que ocorrem na ausência de doença cardíaca estrutural significativa.^4^ Dois arranjos distintos são descritos atualmente.^8^^,^^9^ Ondas J proeminentes, elevação do segmento ST ≥ 2 mm e ondas T negativas em pelo menos uma derivação precordial direita convencional ou superior – o padrão eletrocardiográfico de Brugada do tipo 1 – é a principal característica da síndrome e essencial para diagnóstico, prognóstico e estratificação de risco.^9^ Entretanto, o diagnóstico de SB só é assegurado quando o ECG do tipo 1 está associado a sintomas arrítmicos, histórico familiar de PEBr ou MSC, e outros marcadores específicos.^8^ Caso contrário, o indivíduo é considerado apenas portador do padrão eletrocardiográfico de Brugada. Por outro lado, o padrão tipo 2 ou “em sela” (onda r' seguida por um segmento ST elevado e convexo), embora altamente suspeito, não é diagnóstico e requer investigação suplementar.^8^^,^^9^

Identificar a frequência e distribuição do PEBr é essencial para predição da carga futura da doença e orientação de políticas de saúde pública.^10^ Os aspectos epidemiológicos da SB e dos padrões eletrocardiográficos a ela relacionados foram a questão principal de importantes estudos publicados nas últimas três décadas. No entanto, determinar o ônus desses distúrbios não é tarefa fácil e pode apresentar críticos entraves.

O primeiro diz respeito à natureza transitória do substrato eletrofisiológico de base, que pode ser modulado ou induzido por drogas e alterações autonômicas ou metabólicas.^8^^,^^11^ Outrossim, o PEBr pode ser dinâmico e, por vezes, oculto.^9^ Desta forma, as circunstâncias, a duração, a periodicidade e as ferramentas utilizadas para a aquisição e o monitoramento do ECG podem impactar consideravelmente a probabilidade diagnóstica. Como as alterações elétricas se concentram na via de saída do ventrículo direito, o posicionamento de V1 e V2 nos espaços intercostais superiores aumenta a chance de reconhecimento do PEBr, em comparação com o ECG convencional de 12 derivações.

Outra armadilha diz respeito ao curso clínico. Os portadores de PEBr e a maioria dos pacientes com SB são assintomáticos.^8^ Eles não procuram voluntariamente os serviços médicos e, portanto, podem estar sub-representados em coortes de centros terciários. Além disso, PEBr não é específico da SB, outras doenças devem ser excluídas, como infarto agudo do miocárdio, desequilíbrio eletrolítico, embolia pulmonar e massas mediastinais.^12^

Por último, e não menos importante, a frequência do PEBr e da síndrome de Brugada diferem significativamente entre os continentes. Essa grande variação se deve provavelmente à interação entre aspectos locais/ambientais e raciais/genéticos.^13^ Entretanto, ela pode também refletir a heterogeneidade dos estudos em relação ao tipo/número de centros de pesquisa, métodos de amostragem, características e tamanho da população estudada, critérios de inclusão e ferramentas de triagem. Esses fatores resultam em achados que nem sempre são generalizáveis. Por isso, a epidemiologia da SBr e do PEBr continuam sendo desconhecidas em muitas partes do globo.^14^

O primeiro estudo brasileiro sobre a prevalência do PEBr foi publicado na presente edição dos Arquivos Brasileiros de Cardiologia.^15^ Os autores avaliaram o banco de dados de telemedicina dos laudos de traçados de ECG convencional de 12 derivações de 716.973 indivíduos atendidos nas unidades básicas de saúde de mais de 250 cidades em Santa Catarina, Brasil, entre 2010 e 2015. Nessa amostra, diferente da maioria dos estudos, o tipo 1 (4,6/100.000) foi mais frequente do que o tipo 2 (3,0/100.000).^12^^,^^14^ É interessante notar que a prevalência encontrada pelos autores foi no mínimo dez vezes menor do que a estimada em países ocidentais e menos de 1% daquela descrita na Ásia, onde a SB é endêmica, por estudos que também usaram ECG convencional de 12 derivações.^14^ O estudo de Militz *et al*. é notável pelo tamanho considerável de sua amostra e pela extensa cobertura territorial. Não obstante, se esses valores representam todos os habitantes de Santa Catarina e a sua variação genética é uma questão a ser confirmada.^15^ Ademais, esses números não devem ser extrapolados para toda a população do Brasil, um país de dimensões continentais onde cada estado exibe uma mistura única de raças e origens étnicas. Embora não se possam excluir questões de amostragem e diagnóstico, já que a maioria dos traçados de ECG não foi revista, esse estudo foi o primeiro a avaliar a frequência de PEBr no Brasil. Ainda assim, são necessários mais estudos para delinear a carga da SB e de seus padrões eletrocardiográficos no território nacional.
