# Supplementary material for: Padrão Eletrocardiográfico de Brugada – Dificuldades no Reconhecimento de uma Condição Potencialmente Letal
Source: Arq Bras Cardiol. 2021 Aug 9;117(2):350–1. [Article in Portuguese] doi: 10.36660/abc.20210596 (PMC8395805; doi:10.36660/abc.20210596)
Supplement: Translation

# Translation: Brugada ECG Pattern – A Blip on the Radar for a Pontentially Life-Threatening Condition

**Affiliations:** 1 São PauloSP Brazil Instituto do Coração (InCor) - Hospital das Clínicas HCFMUSP - Faculdade de Medicina da Universidade de São Paulo, São Paulo, SP – Brazil.

**Keywords:** Brugada Syndrome, Brugada Electrocardiographic Pattern, Brugada ECG Prevalence, Standard 12-Lead ECG, Brazil

The finding of a singular ST-segment elevation in the right precordial leads was first considered a normal variant.^1^ An initial hint that this electrocardiographic (ECG) sign and sudden cardiac death (SCD) were possibly associated sprang from a case series published in 1989.^2^ Three years later, Brugada *et al.* reported on eight patients who presented with right bundle branch block, persistent ST-segment elevation in V1-3, and multiple episodes of ventricular fibrillation, a new clinical and ECG syndrome that was later named after them.^3^ Nowadays, Brugada syndrome (BrS) still attracts much interest due to its high prevalence in specific world regions and potential lethality in otherwise healthy young adults.^4^^,^^5^

Accounting for 4% to 12% of all cases of SCD globally and one fifth of those occurring in structurally normal hearts,^6^ BrS is an autosomal dominant cardiac disease, with incomplete sex- and age-related penetrance, caused by dysfunctional sodium channels.^7^^,^^8^ Although it is usually silent, BrS is clinically remarkable for male predominance, manifesting between the third and fifth decades of life.^4^ Affected patients display ECG abnormalities and increased susceptibility to cardiac arrhythmias.^8^ Symptoms, when present, may vary from syncope to SCD depending on the sort and duration of arrhythmic events, which often occur at rest or in vagotonic conditions.^8^

ECG findings include depolarization and repolarization abnormalities in the absence of overt structural heart disease.^4^ Two distinct arrangements are currently described: type 1 and type 2 Brugada ECG pattern (BrEP).^8^^,^^9^ Prominent J waves, upward ST-segment elevation ≥ 2 mm, and negative T waves in at least one standard or superior right precordial lead, the type 1 (“coved”) pattern, is the BrS signature, and it is essential to diagnosis, prognosis, and risk stratification.^9^ However, diagnosis of BrS is only warranted when type 1 ECG is associated with arrhythmic symptoms, family history of BrEP or SCD, and specific surrogate markers.^8^ Otherwise, the individual will be considered merely a carrier of BrEP. Conversely, the type 2 (“saddle-back”) pattern, an r' wave followed by elevated and convex ST-segment, although highly suspicious, is not diagnostic, and it requires supplementary investigation.^8^^,^^9^

Identifying BrEP frequency and distribution is a cornerstone for predicting future disease load and guiding public health policies.^10^ The epidemiology aspects of BrS and related ECG patterns were the primary issue in pivotal studies published in the last three decades. However, determining the burden of these disorders is not easily accomplished; on the contrary, it might pose critical caveats.

The first one regards the primarily transient nature of the underlying electrophysiological substrate, which can be modulated or induced by drugs and autonomic or metabolic conditions.^8^^,^^11^ Likewise, rather than stagnant, the BrEP is dynamic and often concealed.^9^ Consequently, the circumstances, duration, periodicity, and tools used for ECG monitoring may directly impact the diagnostic yield for Brugada ECG signs. As electrical abnormalities concentrate in the right ventricular outflow tract, positioning V1 and V2 in superior intercostal spaces increases the odds of recognizing a BrEP compared to a standard 12-lead ECG.

Another pitfall lies in the clinical course. Carriers of BrEP and most patients with BrS are asymptomatic.^8^ They do not voluntarily seek health care facilities, and they may, therefore, be underrepresented in tertiary center cohorts. Additionally, BrEP is not specific for BrS; other conditions should be excluded, such as acute myocardial infarction, electrolyte imbalances, pulmonary embolism, and mediastinal masses.^12^

Last but not least, the frequencies of BrEP and BrS differ significantly worldwide. This wide range is likely due to the interaction between local/environmental and racial-specific/genetic aspects.^13^ However, it may also reflect the heterogeneity of studies regarding type/number of research centers, sampling methods, study population characteristics and size, inclusion criteria, and screening tools. These factors result in findings that are not always generalizable. Thereby, the epidemiology of BrS and BrEP remains unknown in many parts of the globe.^14^

The first Brazilian study on BrEP was published in the current edition of Arquivos Brasileiros de Cardiologia.^15^ The authors assessed the telemedicine database for written reports of standard 12-lead ECG tracings from 716,973 individuals attended in basic health units of over 250 cities in Santa Catarina, Brazil, between 2010 and 2015. In their sample, unlike most studies, type 1 (4.6/100000) was more frequent than type 2 (3.0/100000).^12^^,^^14^ Interestingly, the prevalence reported therein was at least ten times lower than that estimated in Western countries and less than 1% of that described in Asia, where BrS is endemic, by studies that also used standard 12-lead ECG.^14^ The study by Militz *et al*. is remarkable for its sizeable study population and extensive territorial coverage, but whether those values represent all of Santa Catarina's inhabitants and their genetic variation remains unclear.^15^ Moreover, these findings cannot be extrapolated to the entire population of Brazil, a continent-sized country where each state has a unique blend of races and ethnic origins. Although sampling and diagnostic issues cannot be excluded, as most ECG tracings were not reviewed, this study was the first to assess the frequency of BrEP in Brazil. Still, more studies are needed to outline the burden of BrS and its patterns nationwide.
